# The Thermal and Histological Evaluation of Lingual Frenulum Incision Using Diode Lasers in an Experimental Model

**DOI:** 10.3390/jcm15103567

**Published:** 2026-05-07

**Authors:** Adriana Cátia Mazzoni, Amanda Maria Vieira, Amanda Rafaelly Honório Mandetta, Raquel Agnelli Mesquita-Ferrari, Ricardo Scarparo Navarro, Ana Paula Taboada Sobral, Rodrigo Labat Marcos, Sandra Kalil Bussadori, Lara Jansiski Motta

**Affiliations:** 1 Postgraduation Program in Biophotonic Medicine, Universidade Nove de Julho, São Paulo 01504-000, SP, Brazil; adrianamazzoni@uol.com.br (A.C.M.); amandahonorio@hotmail.com (A.R.H.M.); raquel.mesquita@gmail.com (R.A.M.-F.); labat@uni9.pro.br (R.L.M.); larajmotta@terra.com.br (L.J.M.); 2 Postgraduate Program in Paediatric Dentistry, Universidade Metropolitana de Santos, Santos 11045-002, SP, Brazil; amandavieira.dentista@gmail.com; 3 Postgraduate Program in Dentistry, Universidade Santo Amaro, São Paulo 04829-300, SP, Brazil; ricardosnavarro@gmail.com; 4 Postgraduate Program in Health and Environment, Universidade Metropolitana de Santos, Santos 11045-002, SP, Brazil; anapaula@taboada.com.br

**Keywords:** diode laser, lingual frenulum, tissue repair

## Abstract

**Background/Objectives:** In lingual frenectomy/frenotomy surgeries, thermal surgical instruments are commonly used because of their hemostatic effect, precise cutting, and lack of need for sutures. However, the effects of tissue heating on the biological repair process remain under investigation. This study aimed to evaluate the thermal and histological effects of a surgical lingual frenulum incisions performed with high-power diode lasers at 450 nm (blue) and 980 nm (infrared), compared with a surgical scissor incision (control group). **Methods:** The experimental project was conducted in two phases: first, an ex vivo experiment on porcine tongues using different surgical instruments, with different parameters, with thermal variations recorded using infrared thermography; and second, an in vivo phase using the instruments previously determined in the ex vivo phase. **Results:** Results demonstrated minimal tissue heating in the control group. There was a thermal elevation with the laser instruments, particularly with the 980 nm and 450 nm diode lasers; however, the 450 nm laser showed a high initial temperature increase, but exhibited faster tissue cooling. Thermal and histological analyses performed immediately after the procedure and on the seventh postoperative day showed clinically similar tissue repair across groups, with less disorganization of collagen fiber bundles on day seven in the group treated with the 450 nm laser. **Conclusions:** In conclusion, both diode lasers can be safely employed for lingual frenulum incision when appropriate parameters are used; however, further clinical studies are warranted to establish safe and effective parameters.

## 1. Introduction

Ankyloglossia, also referred to as tongue-tie, is a congenital anomaly characterized by a thick, short, or anteriorly positioned lingual frenulum, which restricts tongue mobility. The lingual frenulum is a dynamic three-dimensional structure composed of embryonic remnant tissue that forms a submucosal fold extending from the ventral surface of the tongue to the floor of the mouth, consisting of fibrodense connective tissue rich in elastic and muscle fibers, adipose cells, and blood vessels, in addition to the fascia of the oral floor [1]. This mobility restriction can interfere with newborn orofacial development and with activities such as suckling, chewing, swallowing, and phonation, underscoring the importance of early diagnosis and appropriate intervention [2].

Clinically, ankyloglossia results from alterations in the insertion or length of the lingual frenulum and may compromise the development and functional balance of the stomatognathic system [3]. Lingual frenectomy/frenotomy is the surgical procedure indicated for the release of the lingual frenulum, aiming to restore the range of tongue motion and improve compromised oral functions. Traditionally, this procedure has been performed using cold-blade instruments such as scalpels or surgical scissors. Although effective, these techniques are frequently associated with greater intraoperative bleeding, difficulty visualizing the surgical area, the need for suturing, longer surgical time, and postoperative discomfort, particularly in pediatric patients [4].

The lingual frenulum acts in coordination with other oral structures to support essential functions, particularly in neonates, in whom its integrity is crucial for effective breastfeeding, considered the gold standard for infant nutrition and craniofacial development [5]. When ankyloglossia interferes with breastfeeding, diagnosis and treatment planning should be conducted through a transdisciplinary approach [6].

For accurate diagnosis and clinical decision-making, validated protocols are recommended. Clinical assessment should include both morphological and functional evaluation of the lingual frenulum. In Brazil, the Bristol Tongue Assessment Tool (BTAT) has been recommended in maternity settings to identify severe cases, combined with direct observation of breastfeeding to assess tongue mobility and feeding efficiency [7]. In addition, clinical consensus statements emphasize that treatment indication should consider the functional impact of ankyloglossia, particularly its effect on breastfeeding, rather than anatomical characteristics alone [8].

Furthermore, anatomical and functional classifications of ankyloglossia have been proposed to support diagnosis and guide treatment decisions, highlighting that the choice of surgical technique and timing of intervention may vary depending on the type and severity of the condition [8,9].

Moreover, recent studies suggest that the use of high-power lasers for incision in frenectomy promotes simultaneous coagulation of blood vessels, providing better hemostatic control, a cleaner surgical field, local disinfection, and reduced surgical trauma [10]. However, results remain heterogeneous because different wavelengths, emission modes, and power parameters are used, making direct comparisons between studies difficult [11]. Among the different types of lasers available, diode lasers have distinguished themselves in clinical dental practice by their versatility, portability, and relatively accessible cost. These devices operate at wavelengths with high affinity for tissue chromophores such as hemoglobin and melanin, facilitating efficient cutting and hemostasis [12].

The interaction between laser radiation and biological tissues depends directly on the wavelength used and the application parameters. Shorter wavelengths, such as a 450 nm diode laser (blue), exhibit higher hemoglobin absorption, potentially enhancing the hemostatic effect. In contrast, longer wavelengths, such as a 980 nm diode laser, are also absorbed by water, promoting greater thermal penetration into tissues. These differences may influence both intraoperative thermal behavior and the tissue healing process [13].

Despite the growing use of high-power diode lasers in soft tissue procedures, there is still limited evidence integrating thermal effects with subsequent biological tissue response under controlled experimental conditions. In particular, studies combining ex vivo thermal analysis with in vivo histological evaluation remain scarce. A better understanding of how different wavelengths influence both heat generation and tissue repair is therefore essential to support safer and more effective clinical protocols, especially in scenarios involving vulnerable populations such as very young infants. To address this gap, the present study adopts a two-phase experimental design, in which ex vivo thermal assessment guides the selection of surgical parameters and instruments for subsequent in vivo evaluation to better approximate clinically relevant conditions.

Although laser-based procedures offer clinical advantages, the thermal effects generated during surgical incision may cause collateral damage, such as excessive temperature increases leading to local tissue carbonization and delayed tissue repair. Therefore, evaluating thermal variation during the procedure is essential to ensure the safety of the technique [14]. Thus, this study aims to evaluate surgical lingual frenulum incision using 450 nm (blue) and 980 nm (infrared) diode lasers, comparing them with Iris surgical scissors, thereby expanding knowledge and informing the formulation of safer, more effective clinical protocols.

## 2. Materials and Methods

### 2.1. Study Design

This was an experimental, controlled, comparative study conducted in two phases: the first phase using ex vivo specimens and the second using an in vivo model. Firstly, sixty ex vivo porcine tongues were used, supplied by the registered butcher shop. To select instruments for the second phase, a comparative analysis was conducted among instruments from the first phase, evaluating the temperatures recorded during thermal instrument use. The Iris surgical scissors were chosen for the control group because they are mechanical instruments that work by cutting and do not promote changes in temperature.

Secondly, we conducted an experimental model with 30 prepubescent Wistar rats of both sexes, aged 22–30 days. The sample size and experimental design were defined based on previously published studies using similar experimental models [15,16,17]. The following instruments were selected: Iris surgical scissors (control group), the high-power pulsed infrared diode laser at 980 nm, 1.3 W, 20 Hz (Thera Lase Surgery™, DMC, São Carlos, Brazil) and the high-power pulsed blue diode laser at 450 nm, 1.5 W, frequency 20 Hz (Thera Blu™, DMC, São Carlos, Brazil), selected based on their distinct optical absorption characteristics and thermal interaction with biological tissues, as described in the literature [13], with particular differences in hemoglobin absorption and tissue penetration. Animals were maintained under controlled conditions of temperature, lighting, and feeding, with free access to water and standard chow, following a pre-established acclimatization period. All experimental procedures were conducted in accordance with the ARRIVE (Animal Research: Reporting of In Vivo Experiments) guidelines for animal research.

### 2.2. Ex Vivo Experiment

As part of the two-phase experimental design, the ex vivo phase included different instruments and parameters to evaluate thermal behavior and support the selection of devices for the subsequent in vivo experiment. Porcine tongues were thawed and maintained at room temperature. Researchers employed all required personal protective equipment (PPE) and biosafety measures to avoid interference with results.

The pieces were distributed in five experimental groups. Each specimen was placed on a tray during incision and was handled with a Dietrich Golgran clinical forceps (Golgran, São Caetano do Sul, Brazil). The temperature was measured and recorded using a thermographic camera (Teledyne FLIR C5, Wilsonville, OR, USA); a standard location on the tray was established, where all specimens remained during each surgical procedure. The incision was performed in the lingual frenulum region, and the generation of local tissue heating caused by the selected instruments was compared. All instruments used the parameters described in Table 1.

### 2.3. In Vivo Experiment

Although the Er:YAG laser was evaluated in the initial ex vivo phase, it was not included in the in vivo experiment due to methodological considerations related to the clinical scenario under investigation. Its use typically requires air–water spray, which limits its applicability in very young infants. In preliminary testing, when the water spray was removed to adapt the device to these conditions, excessive local heating was observed.

A total of 30 animals were randomly distributed into three groups: Group 1—Iris surgical scissors (*n* = 10); Group 2—high-power diode laser at 980 nm (infrared), 1.3 W, 20 Hz (*n* = 10); Group 3—high-power diode laser at 450 nm (blue), 1.5 W, 20 Hz (*n* = 10), hereafter referred to as the scissors group (control), laser 980 nm group, and laser 450 nm group, respectively. Animals were kept at the UNINOVE animal facility under required safety standards. Anesthesia was administered and supervised by the facility’s veterinarian. Rats received intramuscular injections of ketamine hydrochloride (100 mg/kg) combined with xylazine hydrochloride (10 mg/kg). After anesthesia, antisepsis of the lingual frenulum region was performed with saline solution, followed by local infiltrative anesthesia with 2% lidocaine adjusted according to body weight, to prevent animals from suffering or stress and avoid hormone-mediated interference with tissue repair.

**Figure 1:**
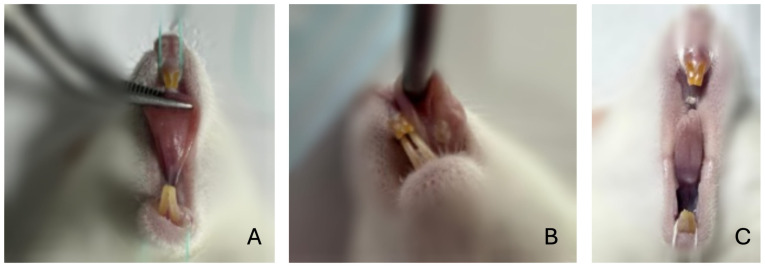
Surgical incision techniques: (**A**) incision performed with surgical scissors; (**B**) incision performed with a 450 nm diode laser; (**C**) incision performed with a 980 nm diode laser.

The tongue was elevated with Dietrich forceps during each procedure to prevent heat transfer from the operator’s hand. The incision dimensions were standardized to approximately 1 mm in diameter and 1 mm in depth in the lingual frenulum region for all experimental groups (Figure 1).

During the experiment, the operator was assisted by another dental surgeon to hold the specimen. Each animal was placed in a standardized location on the tray during the surgical procedure, and room temperature was maintained between 20 °C and 23.5 °C. Thermal variation during incisions was assessed by infrared thermography, using a thermal camera positioned at a standardized distance from all animals, directed at the incised region.

On the day of surgery, tissue samples approximately 1 mm in diameter were collected from the entire surgical site in five animals per group for immediate histological evaluation. These animals were euthanized; the remaining 15 animals (5 per group) were maintained for 7 days for late evaluation and received paracetamol drops dissolved in their drinking water for analgesia. On day 7, a clinical assessment of the tissue repair condition was performed; local tissue heating generated by the instrument was measured during incision using the FLIR C5™ thermographic camera with MSX™ (Multi-Spectral Dynamic Imaging) technology (Teledyne FLIR, Wilsonville, OR, USA). Animals were then euthanized, and additional tissue samples of approximately 1 mm in diameter were collected from the healing site for histological analysis. Euthanasia was performed following the Brazilian Guidelines for Good Practices in Animals.

### 2.4. Histological Analysis

Mucosal samples from all groups were immediately placed in 10% formalin for 24 h for biological tissue processing, following the methods described by Chaves Borges and Mineo (1997) and the World Health Organization (2003) [18,19]. Biological material was sent to the Histocell Private Laboratory™ (São Paulo, Brazil) for technical analyses. Histological slides were subjected to two types of analysis: first, evaluation of inflammatory infiltrate in hematoxylin and eosin (H&E)-stained sections; second, assessment of disorganized collagen fiber bundle concentration and fibrotic tissue in Masson’s trichrome (MT)-stained sections (see Supplementary Materials). Both analyses were performed using an Opticam 0300S microscope (Opticam Microscopy Technology, São Paulo, SP, Brazil) at 4×, 10×, and 40× objectives, corresponding to total magnifications of 40×, 100×, and 400×, respectively.

### 2.5. Data Analysis

Data were analyzed using descriptive and inferential statistics. Thermal evaluation data were expressed as maximum temperature values, thermal variation, and tissue cooling time, and presented as means and standard deviations or medians and interquartile ranges, depending on the data distribution. Histological evaluations were conducted by an examiner blinded to group allocation to minimize bias. The data in this study were analyzed using nonparametric methods due to nonnormality, as confirmed by the Shapiro–Wilk test. Continuous variables were described as medians and interquartile ranges (IQR). Friedman’s test was used to compare initial, final, and cooling temperatures across groups, and Dunn’s test with Bonferroni correction was used for post hoc multiple comparisons. Statistical significance was established with a 95% confidence level (*p* < 0.05). The analysis was carried out using GraphPad Prism™ version 11 (GraphPad Software, San Diego, CA, USA) and jamovi™ version 2.7.6 (The jamovi project, Sydney, Australia).

## 3. Results

### 3.1. Ex Vivo Experiment

We analyzed the use of surgical instruments during incisions in the specimens, each with distinct parameters, yielding 12 groups. The incisions were performed on the ex vivo porcine tongue, and the initial temperature showed no significant differences between the instruments (*p* > 0.05).

It was observed that even with the use of scissors, which are instruments with a cold blade, there was a slight warming at the beginning of the surgical procedure. The authors observed that no instrument, depending on its use, generated heat above 40–42 °C, which is the threshold temperature that could cause thermal damage to the tissue through denaturation and irreversible coagulation of cytoplasmic proteins.

The median initial temperature was lowest for the scissors (5.9 °C) and ranged from 11.4 °C to 11.9 °C for electrosurgical instruments. Among the laser devices, median temperatures ranged from 8.4 °C to 14.0 °C, with higher values observed for the 980 nm and 450 nm diode lasers. Detailed descriptive data, including interquartile ranges and minimum–maximum values, are presented in Table 2.

When the final temperatures of the tongues were compared in relation to the instruments used, there were differences only when comparing scissors to electrocautery (*p* = 0.035), scissors vs. electric scalpel (*p* = 0.041), scissors vs. D-Storm™ continuous high-power diode laser 2.0 W (*p* = 0.021), and scissors vs. D-Storm™ long pulse high-power diode laser 7.0 W (*p* = 0.007).

**Figure 2:**
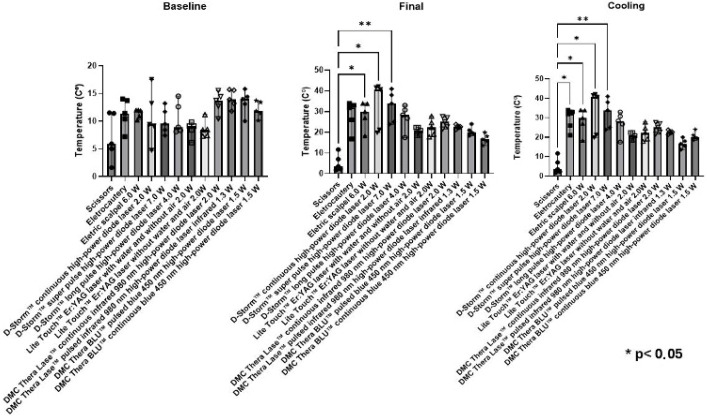
Temperature variation in the ex vivo experiment. The temperature was recorded in °C. Statistical significance is indicated as follows: * *p* < 0.05; ** *p* < 0.01.

Similarly, the cooling temperatures were only different when comparing instruments: scissors vs. electrocautery (*p* = 0.03); scissors vs. electric scalpel power (*p* = 0.0413); scissors vs. D-Storm™ continuous high-power diode laser 2.0 W (*p* = 0.0213); and scissors vs. long pulse D-Storm™ long pulse high-power diode laser 7.0 W (*p* = 0.0075). In the intra-group analysis, the initial–final, initial–cooling, and final–cooling conditions showed no statistically significant differences (Figure 2).

### 3.2. In Vivo Experiment

The in vivo experiment evaluated the clinical, thermal, and histological outcomes of lingual frenulum incisions performed using surgical scissors and high-power diode lasers (450 nm and 980 nm).

During surgical procedures, it was observed that both evaluated wavelengths, 450 nm and 980 nm, enabled effective lingual frenulum incisions. However, behavioral differences in laser-tissue interaction were identified, especially in the immediate thermal response. Thermal analysis showed that both the 980 nm and 450 nm high-power diode lasers caused significantly greater temperature increases at the incision site than surgical scissors. Tissue cooling after the incision was faster with the 450 nm high-power diode laser group (blue) than with the 980 nm high-power diode laser group (infrared).

This suggests a more superficial interaction with soft tissues for the 450 nm high-power diode laser, related to its lower optical penetration depth and reduced tissue heating, since this wavelength demonstrated high absorption by the chromophores melanin and hemoglobin, does not interact with water, which can act as a heat conductor, allowing greater tissue penetration by the 980 nm laser. From a macroscopic perspective, both laser groups created well-defined incisions with adequate lingual frenulum separation and no significant intraoperative bleeding. Hemostasis was achieved with both laser wavelengths, although one animal experienced controlled bleeding with the 980 nm diode laser. In the control group using Iris surgical scissors, excessive bleeding occurred during the surgical procedure in four animals. On clinical day 7, the 450 nm laser group showed a larger area of granulation tissue than the 980 nm high-power diode laser group, whereas the scissors group showed no significant visual changes.

Histologically, the initial inflammatory infiltrate on day zero showed that tissues subjected to the 450 nm high-power diode laser had a larger area of immediate thermal coagulation near the incision line. This zone exhibited more evident structural damage, including protein denaturation and greater collagen fiber compaction.

In contrast, samples from the 980 nm high-power diode laser group showed a more limited area of thermal damage, with clearer preservation of nearby tissue structures. The scissors group displayed a hemorrhagic area at the incision site on day zero.

The inflammatory response in histological sections differed between groups (see Supplementary Figures S1–S12). The 980 nm laser group showed a moderate inflammatory infiltrate in the early phases, consistent with thermal and tissue stimulus from the surgical incision. The 450 nm high-power laser group exhibited a more intense inflammatory reaction immediately after the injury, but over the course of 7 days, it demonstrated less edema and a smaller number of inflammatory cells in the connective tissue near the incision.

Regarding tissue repair, granulation tissue formed in both laser groups, but it differed in features. In the 450 nm laser group, the newly formed tissue showed a larger healing area and greater structural organization, with early remodeling signs observed at day 7, with an even better appearance than that achieved with scissors. In the 980 nm laser group, satisfactory tissue repair also occurred, but the healing area was smaller and more concentrated, with denser collagen deposition and a thicker fibrotic scar on day 7.

## 4. Discussion

Following statistical analysis and comparative evaluation of thermal and histological data, the results suggest that the different lingual frenulum incision techniques evaluated in this study were suitable for the experimental model employed. Both the cold-blade technique using surgical scissors and high-power diode lasers at 450 nm and 980 nm enabled effective lingual frenulum release in Wistar rats.

Lingual frenulum release through surgical incision requires adequate removal of fibers restricting tongue movement, restoring functional tissue mobility without compromising adjacent structures. In experimental models such as Wistar rats, this approach enables a more precise understanding of the thermal and histological effects of different surgical instruments, contributing to the standardization of safer, more effective techniques [20,21].

The results of the present study demonstrated that, regardless of the instrument used, complete lingual frenulum sectioning was achievable, enabling immediate tissue mobility. However, significant differences were observed in thermal behavior and histological repair patterns, particularly when comparing the 450 nm and 980 nm high-power diode lasers with surgical scissors, corroborating prior findings in the literature [11,22,23]. The lower thermal elevation observed in the control group submitted to scissors incision was expected, since mechanical instruments do not directly promote significant heat generation. This technique was associated with greater intraoperative bleeding and absence of immediate hemostasis, factors that may influence clinical patient management, the inflammatory microenvironment, and the initial healing process [1,24].

In contrast, high-power diode lasers induced significant thermal elevations during incision, with the 980 nm wavelength showing greater thermal accumulation than the 450 nm laser. This behavior is directly related to its higher absorption by water and hemoglobin, which may explain the greater thermal accumulation observed in this group [25,26]. The 450 nm high-power diode laser, in turn, produced greater superficial heating and lower thermal accumulation, which was associated with the more favorable histological findings observed in this group [27,28].

Histological analysis performed immediately after the procedure revealed larger zones of thermal coagulation in the laser groups. Nevertheless, at the seventh postoperative day, improved clinical tissue repair was observed in the 450 nm high-power diode laser group, indicating that when used with appropriate safe and effective parameters, diode lasers do not compromise the healing process in the medium term [29,30,31]. The 450 nm high-power diode laser group showed less disorganization and a lower scar tissue density, suggesting a more physiological and balanced tissue repair compared with the 980 nm high-power diode laser group. These findings are consistent with studies reporting that wavelengths with lower penetration depth tend to induce more controlled inflammatory responses [32,33].

These findings are in agreement with previous studies showing that laser–tissue interaction depends not only on wavelength but also on absorption by specific tissue chromophores and on the irradiation parameters used, which together influence thermal effects and tissue organization [34]. In addition, it is important to consider that laser parameters (such as power, emission mode, and exposure time) can be adjusted to modulate tissue response, potentially influencing the healing process.

Excessive fibrotic tissue formation has been associated with thermal damage, elevated local temperature, and exacerbated fibroblast activation. In the present study, although severe fibrosis was not observed, the 980 nm group showed a greater tendency toward tissue disorganization, possibly related to the greater accumulated thermal effect during incision, even when operating in pulsed mode [33,34,35,36].

The use of pulsed mode for both high-power diode lasers was fundamental to minimizing thermal damage and enabling heat-dissipation intervals, a strategy widely recommended in the literature for soft-tissue procedures. The appropriate selection of laser parameters remains essential to control thermal effects and optimize tissue response, as supported by previous studies [12,34,37,38].

The Wistar rat model proved adequate for evaluating the thermal and histological effects of high-power diode lasers, enabling standardized, reproducible analyses. However, biological variability and the experimental model itself may influence the healing process, reinforcing the need for complementary studies [39,40].

The Er:YAG laser was evaluated in the initial ex vivo phase but was not included in the in vivo experiment due to methodological considerations related to the clinical scenario under investigation. The study was designed to simulate lingual frenulum procedures in very young infants using an early developmental animal model. In this context, the use of Er:YAG lasers is limited by the need for air–water spray, which may not be suitable for patients with immature swallowing reflexes. In preliminary testing, when the water spray was removed to adapt the device to these conditions, an increase in local temperature was observed, altering its expected interaction profile. Therefore, its exclusion from the in vivo phase reflects a methodological decision to preserve clinical relevance and experimental consistency.

Taken together, the results of the present study indicate that both high-power diode lasers at wavelengths of 450 nm and 980 nm may be used for lingual frenulum incision when controlled parameters are applied. However, the 450 nm laser demonstrated advantages in terms of thermal control and histological organization, suggesting greater potential for future clinical applications [41,42]. It is important to emphasize, however, the need for finer and more rigid fiber tips to provide adequate support for the laser to perform local incision more accurately and effectively.

Finally, further studies are necessary, particularly clinical trials, to deepen understanding of the biological effects of different wavelengths, evaluate longer follow-up periods, and explore different irradiation protocols to establish more precise and safe clinical protocols for lingual frenulum release. Additionally, this study presents limitations, including the use of an experimental animal model, a relatively short follow-up period, potential variability in tissue healing responses, the absence of an a priori sample size calculation, and its exploratory experimental design, all of which should be considered when interpreting the findings. Future studies with larger sample sizes, longer observation periods, and formal statistical power calculations are recommended to validate and expand upon these results.

## 5. Conclusions

It was possible to observe that thermal instruments promoted comparable patterns of tissue heating and repair in this experimental model of lingual frenulum incision. Although both high-power diode lasers evaluated (450 nm and 980 nm, pulsed mode) showed similar healing outcomes, the 450 nm laser demonstrated potential advantages, including the absence of intraoperative bleeding and a lower presence of disorganized collagen fibers on the seventh postoperative day. This resulted in a healing pattern more similar to the one observed with scissors. These findings suggest potential clinical benefits, including improved surgical field conditions and favorable tissue organization during early healing. Additionally, based on the thermal behavior observed during the ex vivo phase, particularly the lower thermal accumulation, the 450 nm laser may represent a promising alternative for lingual frenulum procedures. However, these findings should be interpreted with caution, as they are derived from an experimental animal model. Further clinical and histological studies are necessary to confirm these results and to establish safe and effective clinical parameters.

## Figures and Tables

**Table 1:** Instrument parameters.

Parameters	Source Type (Laser/LED):									
	Er:YAG Laser—LiteTouch (Light Instruments, Yokneam, Israel), with and without Air–Water Cooling	High-Power Blue Diode Laser—Thera Blue (DMC)		Laser Diodo Infravermelho de Alta Potência—Thera Lasers (DMC)		High-Power Infrared Diode Laser—Laser D-Storm (Light Instruments) Cooling System: Air Cooling Recommended Room Temperature: 20–30 °C			Electrocautery	Electric Scalpel
		Continuous Mode	Pulsed Mode	Continuous Mode	Pulsed Mode	Continuous Mode	Pulsed Mode	Superpulsed Mode	Continuous Mode	Continuous Mode
Wavelength	2940 nm	450 nm	450 nm	980 nm	980 nm	980 nm	980 nm	980 nm	Bipolar	Bipolar
Tip size	Distal spot size at the tip end: 0.2–1.3 mm	600 µm	600 micrometros	600 micrometros	600 micrometros	_	_	_	filamento níquel cromo fino/comp 18 cm	600 nm
Control	Handpiece (laser) and foot pedal (cart system)	Foot pedal	Foot pedal	Foot pedal	Foot pedal	Foot pedal	Foot pedal	Foot pedal	na mão	Foot pedal
Spectral bandwidth	<1 nm	300–5000 nm	300–5000 nm	±460 nm	±460 nm	_	_	_	_	Primary circuit supply voltage: 110/220 V, 60 Hz
Operating mode	Pulsed Mode	Continuous Mode	Pulsed Mode	Continuous Mode	Pulsed Mode	Continuous Mode	Pulsed Mode	Superpulsed mode	Continuous Mode	Secondary circuit voltage: 35 V
Output power	Up to 8.4 W	60 mW	60 mW	9 W +/− 20%	9 W +/− 20%	até 15 W	até 15 W	até 15 W		
Average radiant power [mW]	2 W	1.5 W	1.5 W	1.5–2 W	1–1.5 W	2 W	7 W	4 W	1200 °C (single power setting)	6 W
Peak power [mW]	_	_	_	_	_	_	_	_	_	_
Power density at aperture [W/cm^2^]	_	_	_	_	_	_	_	_	_	_
Beam area at target [cm^2^]	Approximately 1 cm^2^	Approximately 1 cm^2^	Approximately 1 cm^2^	Approximately 1 cm^2^	Approximately 1 cm^2^	Approximately 1 cm^2^	Approximately 1 cm^2^	Approximately 1 cm^2^	Approximately 1 cm^2^	Approximately 1 cm^2^
Irradiance at target [W/cm^2^]	_	_	_	_	_	_	_	_	_	_
Duty cycle (%) (for pulsed sources)	_	_	_	_	_	_	_	_	_	_
Effective exposure time [s]	_	_	_	_	_	_	_	_	_	_
Exposure time [s]	_	_	_	_	_	_	_	_	_	_
Pulse duration [s]	_	_	90–95 s		90–95 s			25 μs–10 ms		
Pulse interval [s]	_	_	_	_	5.0 ms	_	_	_	_	_
Pulse length	_	_	_	_	10–100 ms		50 Hz–8 Hz	50 Hz–8 Hz		
Period [s]	_	_	_	_	_	_	_	_	_	_
Pulse repetition frequency (for pulsed sources)	10–50 Hz	_	_	_	_	_	_	_	_	60 Hz Radiofrequency
Frequency	20 Hz	_	10 Hz	_	10 Hz	_	8 Hz	8 Hz	_	−/~ 7 MHz
Radiant exposure [J/cm^2^]										
Energy density at aperture [J/cm^2^]										
Radiant energy [J]										
Energy per pulse [J]										
Application technique	Non-contact mode, with the laser beam directed onto the target surface	Contact mode	Contact mode	Contact mode	Contact mode	Contact mode	Contact mode	Contact mode	Contact mode	Contact mode

**Table 2:** Initial temperature variation (°C) according to the surgical instrument and parameters used.

Instrument/Device	Parameters	Median (°C)	IQR (°C)	Min–Max (°C)
Scissors	—	5.9	6.40	1.60–11.50
Electrocautery	—	11.4	3.30	7.20–14.00
Electric scalpel	—	11.9	1.11	10.20–12.03
D-Storm™ diode laser (continuous)	2.0 W	9.4	4.10	4.70–17.50
D-Storm™ diode laser (long pulse)	7.0 W	9.5	3.30	7.40–13.20
D-Storm™ diode laser (super pulse)	4 W/8 Hz/25 s	8.9	4.30	8.10–14.50
LiteTouch™ Er:YAG (with water, no air)	100 mJ/20 Hz/2.0 W	9.1	0.70	6.10–9.80
LiteTouch™ Er:YAG (no water/air)	100 mJ/20 Hz/2.0 W	8.4	1.10	7.30–11.20
Thera Lase™ 980 nm diode (continuous)	2.0 W	13.7	1.70	10.40–15.50
Thera Lase™ 980 nm diode (pulsed)	1.3 W/20 Hz	13.9	2.40	11.80–15.70
Thera BLU™ 450 nm diode (pulsed)	1.5 W/20 Hz	14.0	1.30	10.00–15.80
Thera BLU™ 450 nm diode (continuous)	1.5 W	11.8	2.00	10.10–13.70

IQR: interquartile range.

## Data Availability

The original contributions presented in this study are included in the article and Supplementary Materials. Further inquiries can be directed to the corresponding author.

## Supplementary Materials

The following supporting information can be downloaded at: https://www.mdpi.com/article/10.3390/jcm15103567/s1, Figures S1–S12: Histological analysis of lingual frenulum samples, illustrating the microscopic characteristics observed in the analyzed specimens.

## Abbreviations

ARRIVE	Animal Research: Reporting of In Vivo Experiments
PPE	Personal Protective Equipment
UNINOVE	Universidade Nove de Julho
H&E	Hematoxylin and Eosin
