# Supplementary material for: The Thermal and Histological Evaluation of Lingual Frenulum Incision Using Diode Lasers in an Experimental Model
Source: J Clin Med. 2026 May 7;15(10):3567. doi: 10.3390/jcm15103567 (PMC13206873; doi:10.3390/jcm15103567)
Supplement: Supplementary file 1 [file jcm-15-03567-s001.zip › jcm-4262259-supplementary.pdf]

Histological Analysis of Lingual Frenulum Samples

Tissue samples obtained from the lingual frenulum region were processed for histological analysis. Sections were stained with hematoxylin and eosin (H&E) and examined under an optical microscope (Opticam O300) using 4×, 10×, and 40× objectives, corresponding to total magnifications of 40×, 100×, and 400×.

Supplementary Figure S1
Control Group – Iris Scissors – Day 0

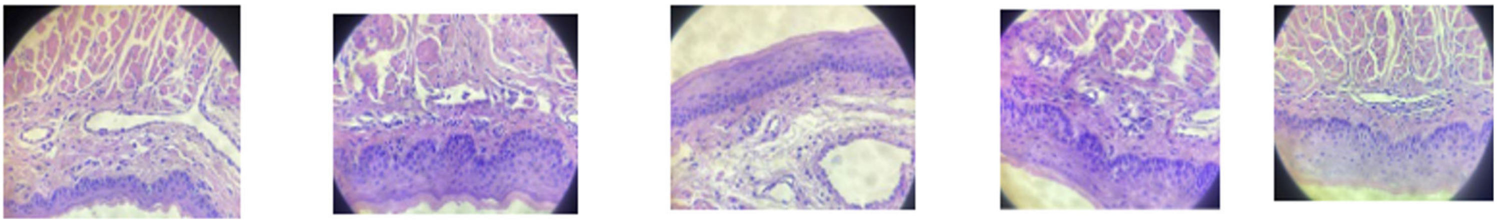

A B C D E

Sample A. Presence of mild diffuse extracellular edema. Preservation of the integrity of the superficial epithelium. Discrete angiogenesis with vascular ectasia. Rare monocytes, plasma cells, and small lymphocytes.

Sample B. Presence of mild extracellular edema. Preservation of the integrity of the superficial epithelium, with slight acanthosis. Discrete angiogenesis. Presence of a few plasma cells and discrete monocytes, histiocytes, and small lymphocytes.

Sample C. Presence of stromal tissue with moderate extracellular edema. Preservation of the integrity of the superficial epithelium. Presence of angiogenesis and a discrete hemorrhagic focus. Presence of a few plasma cells, monocytes, histiocytes, and small lymphocytes.

Sample D. Presence of mild extracellular edema. Preservation of the superficial epithelium with acanthosis. Presence of discrete angiogenesis, mild vascular ectasia, and reactive tissue. Presence of discrete monocytes, histiocytes, and small lymphocytes, with rare mast cells.

Sample E. Preservation of epithelial integrity with acanthosis. Presence of discrete reactive tissue. Presence of monocytes, plasma cells, histiocytes, and small lymphocytes infiltrating the superficial muscular layer.

Supplementary Figure S2

Control Group – Iris Scissors – Day 7

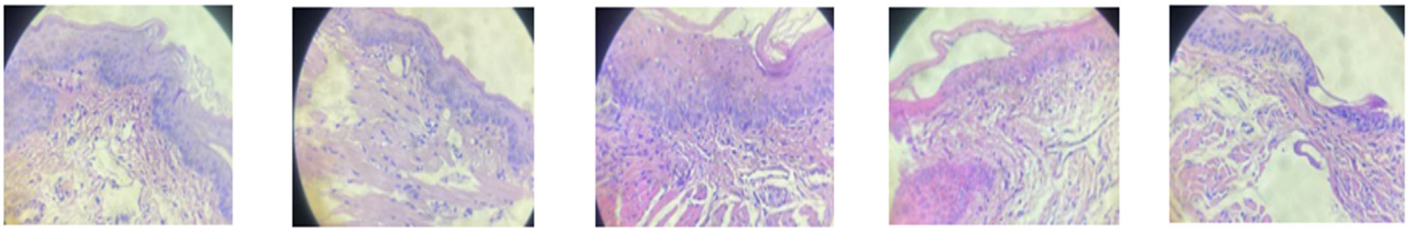

A B C D E

Sample A. Presence of moderate and diffuse extracellular edema in the stroma. Preservation of the integrity of the superficial epithelium. Discrete angiogenesis. Presence of a few monocytes, plasma cells, and small lymphocytes.

Sample B. Presence of mild extracellular edema. Preservation of the integrity of the superficial epithelium. Presence of angiogenesis and vascular ectasia. Presence of a few plasma cells and discrete monocytes, histiocytes, and small lymphocytes, with rare mast cells.

Sample C. Presence of stromal tissue with mild extracellular edema. Preservation of the superficial epithelium with acanthosis. Discrete angiogenesis. Presence of a few plasma cells, monocytes, and small lymphocytes.

Sample D. Presence of stromal tissue with moderate extracellular edema. Relative preservation of the superficial epithelium with acanthosis. Discrete angiogenesis. Presence of congested blood vessels and stromal reactivity. Presence of a few plasma cells, monocytes, and histiocytes.

Sample E. Presence of focal partial loss of superficial epithelial integrity with acanthosis. Presence of discrete reactive tissue and marked extracellular edema. Presence of monocytes, plasma cells, histiocytes, and small lymphocytes, with rare mast cells and mild diffuse infiltration in the muscular layer.

Supplementary Figure S3

Diode Laser Group – 980 nm – Day 0

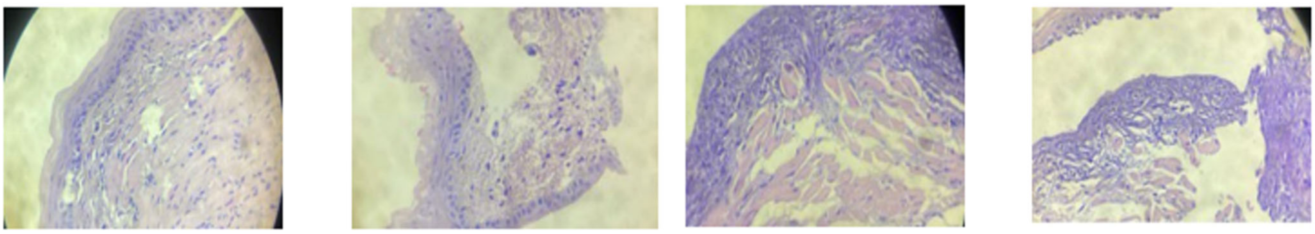

A B C D

Sample A. Presence of stromal tissue with mild extracellular edema. Moderate preservation of the superficial epithelium. Discrete number of neutrophils. Discrete presence of monocytes and small lymphocytes.

Sample B. Presence of stromal tissue with mild extracellular edema and focal areas of degeneration. Presence of discrete superficial hemorrhage. Discrete number of neutrophils. Discrete presence of monocytes and small lymphocytes.

Sample C. Presence of stromal tissue with marked extracellular edema and an extensive focus of tissue degeneration. Discrete focus with preservation of the superficial epithelium. Discrete number of neutrophils. Discrete presence of monocytes and small lymphocytes.

Sample D. Presence of stromal tissue with moderate edema and focal tissue degeneration. Discrete areas with preservation of the superficial epithelium. Discrete number of neutrophils. Discrete presence of monocytes and small lymphocytes.

Supplementary Figure S4

Diode Laser group – 980 nm – Day 7

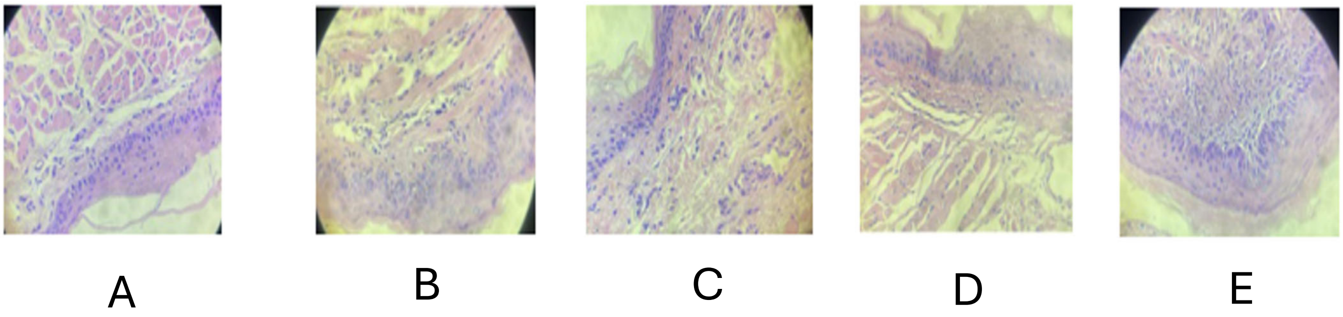

Sample A. Presence of stromal tissue with moderate and diffuse extracellular edema. Preservation of the superficial epithelium with mild acanthosis. Discrete angiogenesis. Presence of rare monocytes and small lymphocytes.

Sample B. Presence of stromal tissue with moderate and diffuse extracellular edema. Preservation of the superficial epithelium with mild acanthosis. Discrete angiogenesis. Presence of rare mast cells and discrete monocytes and small lymphocytes.

Sample C. Presence of stromal tissue with moderate and diffuse extracellular edema. Preservation of the superficial epithelium with acanthosis. Discrete angiogenesis and reactive tissue. Presence of rare neutrophils and discrete monocytes, histiocytes, and small lymphocytes.

Sample D. Presence of stromal tissue with moderate and diffuse extracellular edema. Preservation of the superficial epithelium with acanthosis. Discrete angiogenesis and vascular ectasia with reactive tissue. Presence of discrete monocytes, histiocytes, and small lymphocytes.

Sample E. Presence of stromal tissue with mild and diffuse extracellular edema. Preservation of the superficial epithelium with acanthosis. Presence of discrete reactive tissue and superficial foci of degeneration. Presence of monocytes, histiocytes, and small lymphocytes.

Supplementary Figure S5

Diode Laser group – 450 nm – Day 0

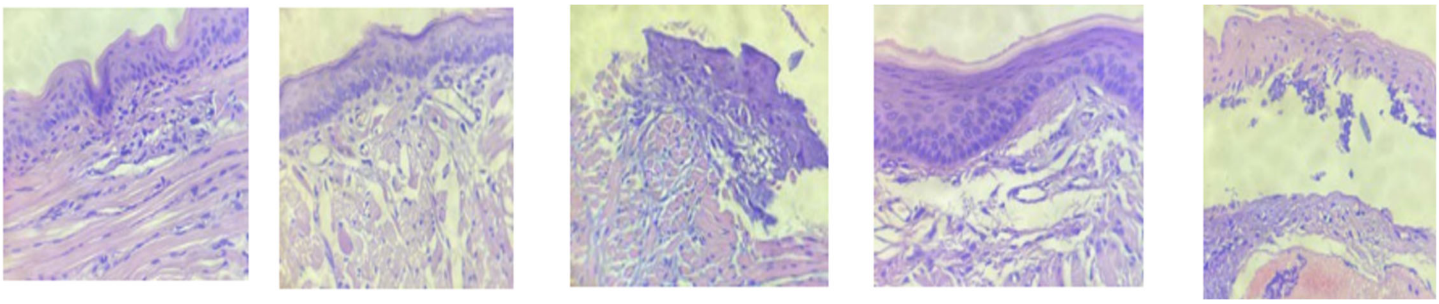

A B C D E

Sample A. Presence of mild extracellular edema in the stroma. Preservation of the integrity of the superficial epithelium. Discrete angiogenesis. Presence of a few monocytes, plasma cells, and small lymphocytes, with rare mast cells.

Sample B. Presence of mild to moderate diffuse extracellular edema in the stroma. Preservation of the integrity of the superficial epithelium. Presence of angiogenesis and vascular ectasia. Presence of plasma cells and monocytes/macrophages, as well as small lymphocytes and rare dispersed mast cells.

Sample C. Presence of stromal tissue with extracellular edema and focal tissue degeneration. Diffuse loss of epithelial preservation with disruption of the superficial epithelium. Discrete angiogenesis. Presence of a few plasma cells, monocytes/macrophages, and small lymphocytes, with scattered neutrophils.

Sample D. Presence of stromal tissue with moderate diffuse extracellular edema. Relative preservation of the superficial epithelium with acanthosis. Presence of discrete angiogenesis, dilated/ectatic vessels, and stromal reactivity. Presence of a few plasma cells, monocytes, and small lymphocytes, with rare mast cells.

Sample E. Presence of foci with partial loss of superficial epithelial integrity and tissue degeneration. Presence of discrete reactive tissue with extracellular edema. Angiogenesis and vascular congestion observed. Presence of monocytes, plasma cells, histiocytes, and small lymphocytes, with rare mast cells.

Supplementary Figure S6

Diode Laser group – 450 nm – Day 7

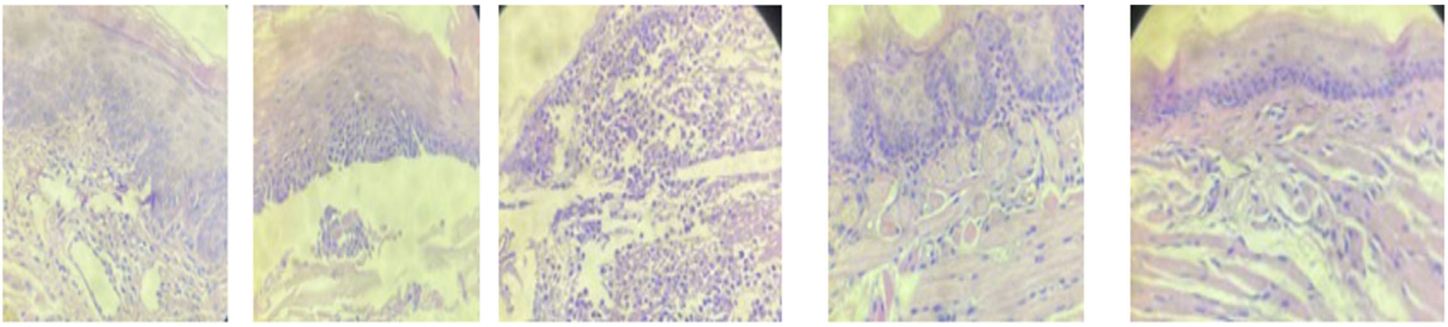

A B C D E

Sample A. Presence of mild extracellular edema in the stroma. Preservation of the integrity of the superficial epithelium with acanthosis. Discrete angiogenesis and vascular dilation. Presence of a few monocytes, plasma cells, and small lymphocytes, with rare neutrophils.

Sample B. Presence of mild to moderate diffuse extracellular edema in the stroma. Preservation of the integrity of the superficial epithelium with acanthosis. Presence of monocytes/macrophages and small lymphocytes.

Sample C. Presence of stromal tissue with extracellular edema and focal areas of tissue degeneration. Preservation of the integrity of the superficial epithelium with marked acanthosis. Discrete angiogenesis with vascular dilation. Presence of plasma cells, monocytes/macrophages, and small lymphocytes, as well as neutrophils and degenerated cells.

Sample D. Presence of stromal tissue with mild to moderate diffuse extracellular edema. Relative preservation of the superficial epithelium with acanthosis. Discrete angiogenesis and mild stromal reactivity. Presence of plasma cells, monocytes/macrophages, and small lymphocytes.

Sample E. Relative preservation of the superficial epithelium. Presence of mild extracellular edema. Presence of monocytes, plasma cells, and small lymphocytes.

Masson's Trichrome Staining

Collagen Deposition Analysis

Tissue samples obtained from the lingual frenulum region were processed for histological analysis. Sections were stained with Masson's Trichrome (MT) to evaluate collagen fiber deposition and organization.

Histological sections were examined under an optical microscope (Opticam O300) using 4×, 10×, and 40× objectives, corresponding to total magnifications of 40×, 100×, and 400×.

Masson's Trichrome staining highlights collagen fibers, which typically appear blue or green, while other tissue components are stained in contrasting colors. Other trichrome staining methods, such as Van Gieson, may also be used to identify collagen fibers, which appear red with that technique. Another commonly used method for collagen detection is Picrosirius Red staining, which stains collagen fibers red and allows evaluation under both bright-field and polarized light microscopy.

Supplementary Figure S7

Control group (iris scissors) – Day 0

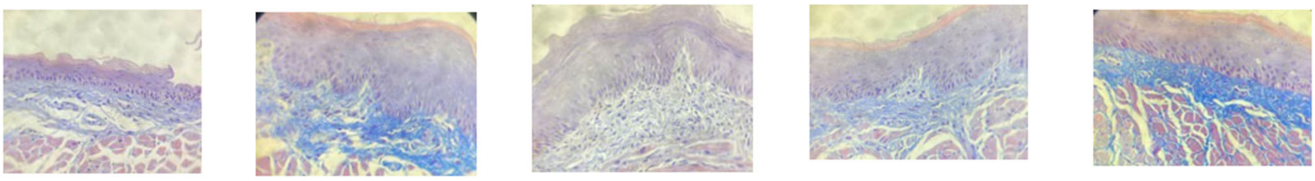

1 1 1 1 1

Supplementary Figure S8

Control group – Iris scissors – Day 7

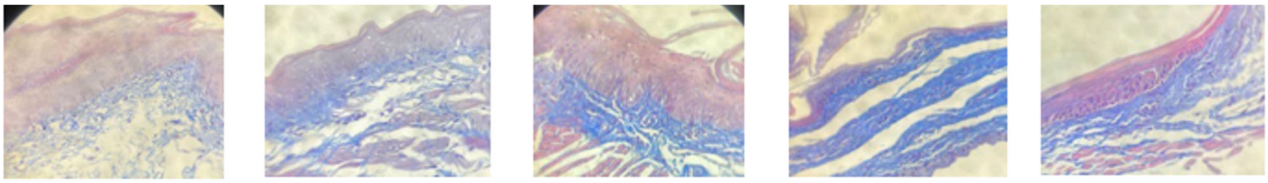

1 1 1 2 1

Supplementary Figure S9

980 nm diode laser group – Day 0

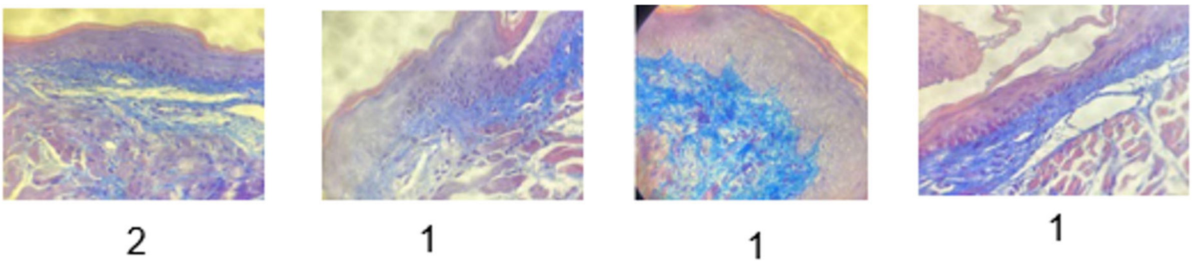

Supplementary Figure S10

980 nm diode laser group – Day 7

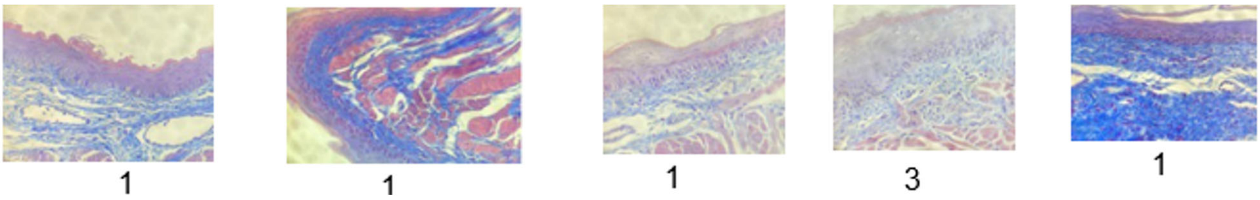

Supplementary Figure S11

450 nm diode laser group – Day 0

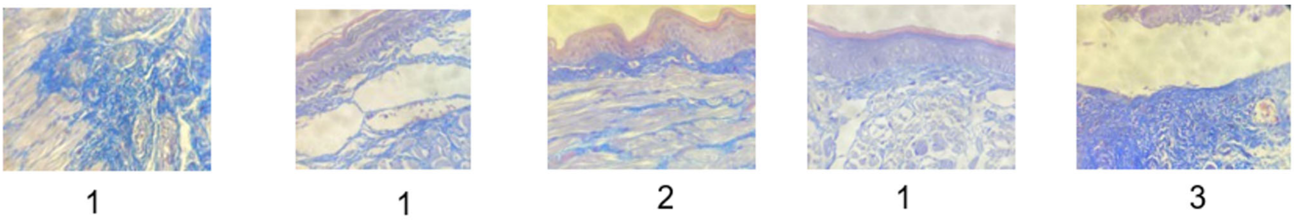

Supplementary Figure S12

450 nm diode laser group – Day 7

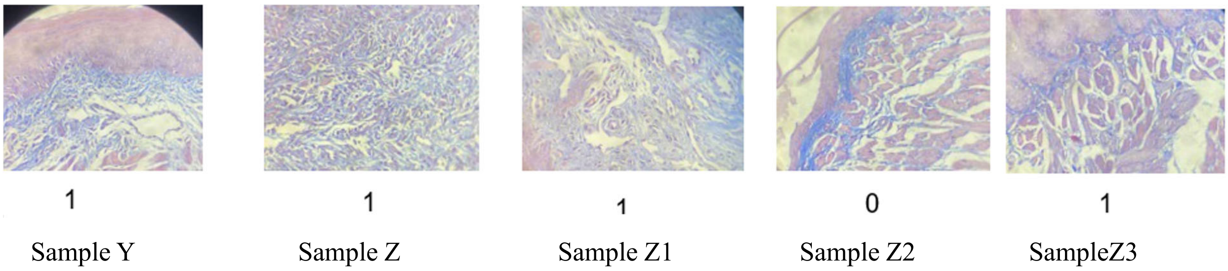

Sample Y

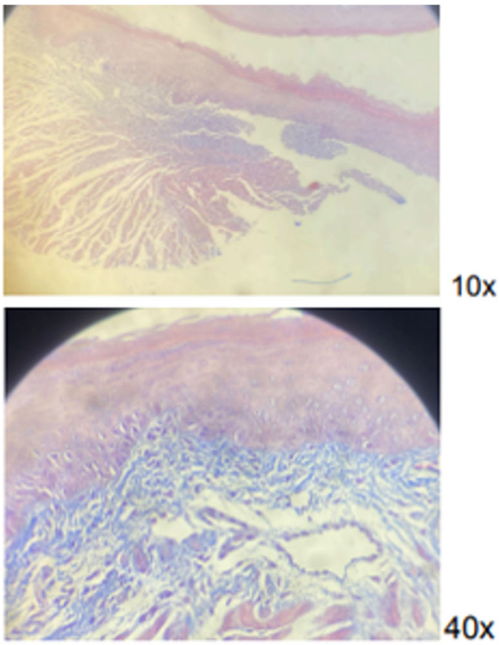

Sample Z

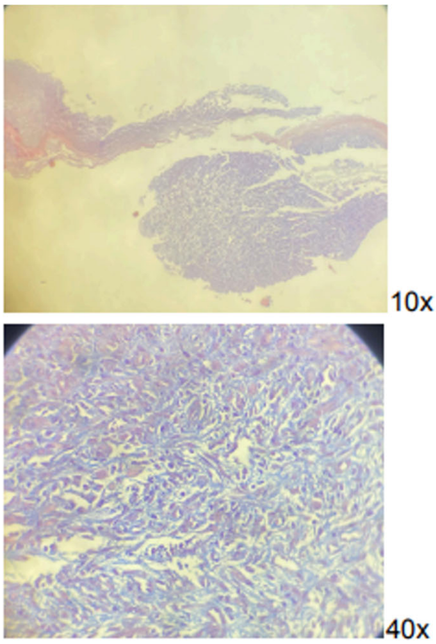

Sample Z1

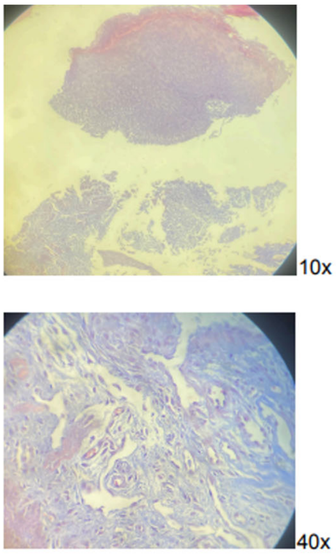

Sample Z2

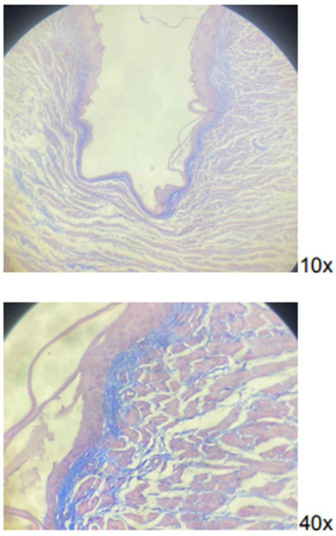

Sample Z3

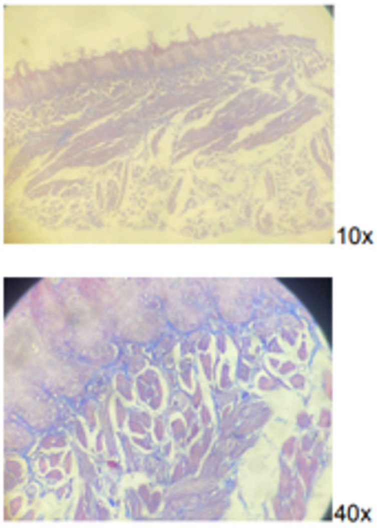

Note: To ensure greater precision and standardization in identifying the area of interest in the samples from the seventh day of the 450 nm diode laser group, the magnification used during analysis was adjusted accordingly. Histological sections obtained from the lingual frenulum region and stained with Masson's Trichrome (MT) were examined under an optical microscope (Opticam O300) using 4×, 10×, and 40× objectives, corresponding to total magnifications of 40×, 100×, and 400×, as described above for comparison purposes.
